# Outcome following surgery for colorectal cancer: analysis by hospital after adjustment for case-mix and deprivation

**DOI:** 10.1038/sj.bjc.6600120

**Published:** 2002-02-01

**Authors:** C S McArdle, D J Hole

**Affiliations:** University Department of Surgery, Royal Infirmary, Alexandra Parade, Glasgow G31 2ER, UK; Department of Public Health, University of Glasgow, Glasgow G12 8RZ, UK

**Keywords:** colorectal cancer, survival, case-mix, deprivation, hospital

## Abstract

Outcome, adjusted for case-mix and deprivation, in 3200 patients undergoing resection for colorectal cancer in 11 hospitals in Central Scotland between 1991 and 1994 was studied. There were significant differences among individual hospitals in the proportion of elderly (*P*<0.001) and deprived (*P*<0.0001) patients, the mode (*P*=0.007) and stage (*P*<0.0001) at presentation, and the proportion of patients who underwent apparently curative resection (*P*<0.001). There were no significant differences in postoperative mortality. Cancer-specific survival at 5 years following apparently curative resection varied from 59 to 76%; cancer-specific survival at 2 years following palliative resection varied from 22 to 44%. The corresponding hazard ratios, adjusted for the above prognostic factors, for patients undergoing apparently curative resection varied among hospitals from 0.58 to 1.32; and the ratios for palliative resection varied from 0.73 to 1.26. This study demonstrates that, after adjustment for variations in case-mix and deprivation, significant differences in outcome among hospitals following resection for colorectal cancer persist.

*British Journal of Cancer* (2002) **86**, 331–335. DOI: 10.1038/sj/bjc/6600120 www.bjcancer.com

© 2002 The Cancer Research Campaign

## 

Colorectal cancer is the second commonest cause of cancer death in the Westernized World. Many patients have evidence of locally advanced or metastatic disease at the time of initial presentation; even in those undergoing apparently curative resection, only half survive 5 years.

Previous studies have highlighted apparent differences in outcome among individual surgeons and hospitals (McArdle and Hole, 1991; Hermanek *et al*, 1995). Most of these studies were small, some reported differences in immediate postoperative morbidity and mortality without taking survival into account and most were not adjusted for differences in case-mix. Furthermore none took deprivation, which has recently been shown to be an important prognostic factor (Kogevinas and Porta, 1997; Coleman *et al*, 1999) into account.

The aim of the present study was to establish whether, having adjusted for case-mix and known prognostic factors in patients undergoing surgery for colorectal cancer, significant differences among individual hospitals persist.

## MATERIALS AND METHODS

Patients (3200) who underwent resection for colorectal cancer between 1st January 1991 and 31st December 1994 in 11 hospitals (five University teaching hospitals and six district general hospitals) in the central belt of Scotland were included in the study. Information was abstracted from casenotes by two specially trained data managers. Data for 1991 and 1992 were collected retrospectively; data for 1993 and 1994 were collected prospectively. Details included age, sex, postcode, mode of presentation, site of tumour, extent of tumour spread, the nature of surgery, post-operative mortality, Dukes staging and adjuvant therapy.

The extent of deprivation was defined using the Carstairs Index (Carstairs and Morris, 1991), an area-based measure derived from the 1991 census data, based on the postcode of residents at diagnosis. Carstairs divides the scores into a seven-point scale ranging from most affluent (category 1) to most deprived (category 7).

Tumours were classified according to site; lesions of the caecum, ascending colon and hepatic flexure were classified as right-sided lesions, whereas lesions of the transverse colon, splenic flexure and descending colon were classified as left-sided lesions. Carcinomas arising at the rectosigmoid junction were classified as rectal cancers. The extent of tumour spread was assessed by conventional Dukes' classification based on histological examination of the resected specimen.

Patients were deemed to have had a curative resection if the surgeon considered that there was no macroscopic residual tumour once resection had been completed. Patients with distant metastases who underwent resection or in whom inadequate local clearance was achieved were deemed to have had a palliative resection.

Information on date and cause of death was checked with that received by the cancer registration system through linkage with the Registrar General (Scotland). All patients have been followed for a minimum of 5 years.

Comparisons between hospitals in relation to age, sex, deprivation category, mode of presentation, site of tumour, extent of tumour spread, the nature of surgery, post-operative mortality, Dukes staging and adjuvant therapy were carried out using analysis of variance or X^2^ tests for trend where appropriate.

The percentages of patients surviving 2 and 5 years were calculated using the Kaplan–Meier technique (Kaplan and Meier, 1958). Overall survival includes both cancer-specific and intercurrent deaths, unadjusted for age and sex. To compare survival in patients treated in different hospitals, while taking into account patients' characteristics at presentation, a standard two-step approach was used. Firstly, significant prognostic factors for survival were entered into Cox's proportional hazards model by forward stepwise addition without reference to which hospital the patient had attended (Cox, 1972). Secondly, each hospital was compared with all the others combined by Cox's proportional hazards model, incorporating the identified statistically significant prognostic factors. The model produces as a measure of outcome the relative hazard ratio, which indicates time specific mortality for the selected hospital compared with that for all other hospitals combined. Values greater than one indicate a higher mortality than average. The procedure was carried out separately for patients undergoing curative resection, palliative resection and for all patients combined.

## RESULTS

Of the 3200 patients included in the analysis, 35.1% were aged 75 or over, 19.2% were socio-economically deprived, 30.8% presented as an emergency and 14.9% had evidence of metastatic spread at the time of surgery. Two thousand, two hundred and thirty-five (69.8%) patients underwent apparently curative resection and 965 palliative resection. Postoperative mortality was 4.3% following curative resection and 9.8% after palliative resection; postoperative mortality was 3.7% in those who presented electively and 11.3% in those who presented as an emergency. 2.3% received adjuvant radiotherapy; 2.6% received adjuvant 5-fluorouracil based chemotherapy.

There were 2108 deaths. Overall survival at 5 years was 40%. Fifty-two per cent of those undergoing apparently curative resection survived 5 years and 26% of those undergoing palliative resection survived 2 years. Forty-six per cent of those treated electively survived 5 years compared to only 28% of those who presented as an emergency.

Cancer-specific survival after apparently curative resection was 66% at 5 years; survival for patients with Dukes' A, B and C tumours was 87, 74 and 47% respectively. Cancer-specific survival following palliative resection was 29% at 2 years and 15% at 5 years.

### Differences between hospitals

The number of patients treated in each hospital varied from 173 to 512 (Table 1

**Table 1:**
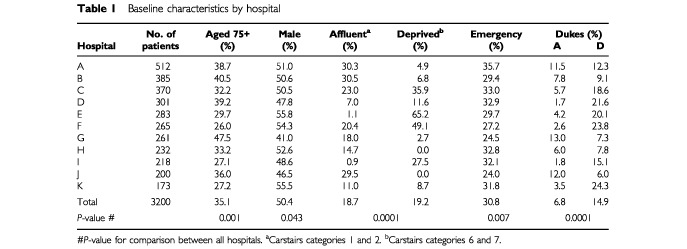
Baseline characteristics by hospital

). The proportion of elderly (75 years) patients varied among hospitals from 26.0 to 47.5%, the proportion of affluent and deprived patients from 0.9 to 30.5% and 0 to 65.2% respectively, and the proportion presenting as an emergency from 24.0 to 35.7%. There was no difference in the distribution of tumours by site between hospitals. The proportion of patients with Dukes' A tumours varied among hospitals from 1.7 to 13.0% and the proportion with evidence of metastatic spread at the time of presentation from 6.0 to 24.3%.

The proportion of patients who underwent apparently curative resection varied among hospitals from 57 to 78% (*P*<0.001). Postoperative mortality in patients undergoing apparently curative resection varied from 2.4 to 7.0% (*P*=0.67) and from 2.4 to 14.6% (*P*=0.46) for those undergoing palliative resection; postoperative mortality varied from 1.6 to 6.1% (*P*=0.56) in those presenting electively and from 6.3 to 18.8% (*P*=0.55) in those presenting as an emergency. The proportion of patients who received adjuvant radiotherapy varied from 0 to 7.6%; the proportion who received adjuvant chemotherapy varied from 0 to 7.5%.

Overall, the proportion of patients who survived 5 years varied among hospitals from 35 to 48%; cancer-specific survival varied from 45 to 62% (Table 2

**Table 2:**
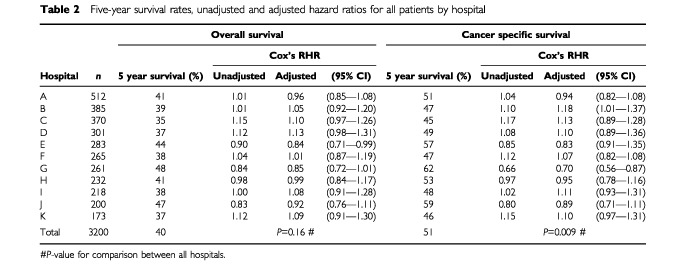
Five-year survival rates, unadjusted and adjusted hazard ratios for all patients by hospital

). Overall, the proportion who survived 5 years following curative resection varied from 44 to 58%; cancer-specific survival varied from 59 to 76% (Table 3

**Table 3:**
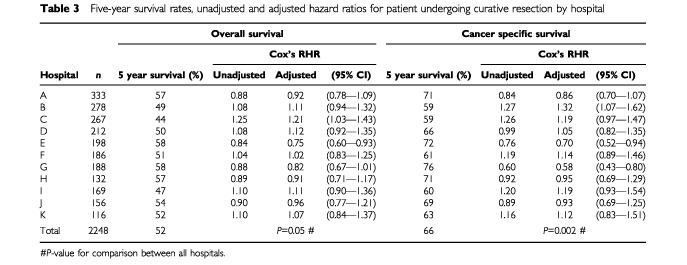
Five-year survival rates, unadjusted and adjusted hazard ratios for patient undergoing curative resection by hospital

). Overall, the proportion who survived 2 years following palliative resection varied from 19 to 37%; cancer-specific survival varied from 22 to 44% (Table 4

**Table 4:**
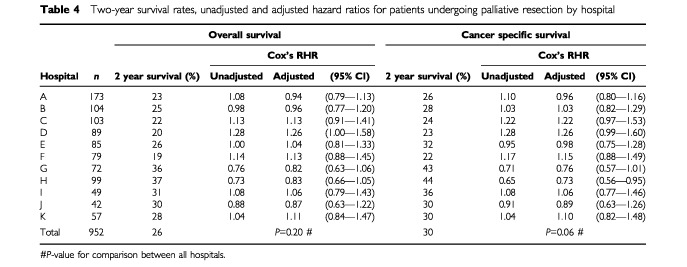
Two-year survival rates, unadjusted and adjusted hazard ratios for patients undergoing palliative resection by hospital

).

**Figure 1:**
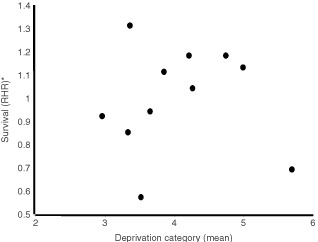
Survival by hospital and deprivation category for patients undergoing a curative resection. *Adjusted for age, sex, mode of presentation, Dukes stage, but not deprivation.

On multivariate analysis, the following factors were shown to significantly influence survival following curative resection – age, sex, deprivation, mode of presentation and Dukes' stage. The hazard ratios for each hospital, adjusted for the above factors, for patients undergoing curative and palliative resection are given in Tables 3 and 4 respectively. The adjusted ratios for patients undergoing curative resection varied among hospitals from 0.58 to 1.32 (cancer-specific survival); the corresponding ratios for palliative resection varied from 0.73 to 1.26. There were no significant differences in survival for either curative or palliative resections between the teaching hospitals and the district general hospitals. The adjusted hazard ratios for each hospital in relation to deprivation are illustrated in Figure 1

.

## DISCUSSION

Currently there is intense interest in comparing outcome following surgery for a variety of common solid tumours in different hospitals. However, such comparisons are usually based on relatively crude registry-based data corrected for age and sex but not for variations in case-mix or deprivation.

The results of the present study confirm that there were large differences among hospitals in age distribution, extent of deprivation, mode of presentation, extent of tumour spread at presentation and pathological stage. There were also differences in the proportion of patients undergoing apparently curative as opposed to palliative resection. There were no significant differences in postoperative mortality rates among hospitals. Differences in cancer-specific survival are likely to reflect variations in the quality of treatment, whereas overall survival includes intercurrent deaths. It is therefore of interest that after adjustment for the above differences in case-mix and type of surgery, significant differences in cancer-specific survival following apparently curative resection among individual hospitals persisted.

The majority of hospitals had similar case-mix and therefore adjustment for the above factors had little impact on the hazard ratios. However, some hospitals had a skewed case-mix. For example in Table 2, hospitals A and I had similar unadjusted cancer-specific survival (RHR=1.04 and 1.02 respectively). Following adjustment, the hazard ratios diverged markedly (RHR=0.94 and 1.11 respectively).

However, it is important to recognize that these analyzes are complex and the results must be interpreted with caution. Despite our endeavours, there are still a number of factors including the assessment of cure and the quality of pathological reporting which may have introduced bias. For example, since it was uncommon to perform tumour bed biopsies, the decision as to whether a resection was curative or palliative was based on the surgeons' subjective impression at the time of surgery. In patients in whom the adequacy of resection was borderline, an optimistic surgeon might believe that he had achieved a cure, whereas a more pessimistic surgeon might assume that he had merely achieved palliation. Depending on how the surgeon perceived the operation, his survival rates following surgery might appear to be better or worse than average.

For example, overall, 30% of patients underwent palliative resection. However, 43% of the resections undertaken in hospital H were deemed to be palliative in nature. Overall, the cancer specific survival for patients undergoing palliative resection was 29% at 2 years and 15% at 5 years. Since the corresponding cancer specific survival rates following palliative resection in hospital H were 44% at 2 years and 28% at 5 years, it is likely that a proportion of the latter patients were wrongly classified as having had a palliative resection. The ‘true’ hazard ratio for this hospital is therefore probably higher than that calculated.

Furthermore, in an era before total mesorectal excision (MacFarlane *et al*, 1993) and detailed examination of the lateral resection margins (Adam *et al*, 1994) were standard practice, variations in outcome may have reflected not only differences among surgeons, but also variations in the quality of pathological reporting. Failure to sample the lateral resection margins or limited sampling of the lymph nodes might lead the pathologist to believe that the lymph nodes and the lateral resection margins were clear of tumour, whilst more rigorous sampling might have revealed the presence of more extensive disease. The resultant pathological stage migration (Feinstein *et al*, 1985) might therefore alter expectation and perhaps outcome.

Despite the above reservations, it would appear that three hospitals had survival rates that appeared to be either significantly better or worse than average. Outcome, relative to deprivation, appeared to be consistent within eight hospitals. One hospital (hospital G) had a lower hazard ratio than other hospitals with a similar case-mix from the same health board. However, both the ratio of intercurrent to tumour related deaths, as evidenced by the magnitude of the difference between the adjusted hazard ratios for overall (RHR=0.82) and cancer-specific (RHR=0.58) survival, and the survival rate following palliative resection were higher than comparable hospitals. Both these factors are likely to have contributed to stage migration and it is therefore probable that the ‘true’ hazard ratio was higher, i.e. less significant, than that calculated.

It is of particular interest that one hospital (hospital B) with a relatively favourable case-mix had significantly poorer survival rates. In contrast, one hospital (hospital E) with a high proportion of deprived patients presenting with advanced disease, nevertheless achieved higher survival rates. Compared to the predicted mortality and adjusting for case-mix, this equates to an excess mortality of approximately 25% in hospital B and a reduction of approximately 25% in the number of patients dying within 5 years of a curative resection in hospital E.

There are two possible explanations for the differences in outcome among hospitals, namely the number of patients treated at each hospital and whether the surgeons were specialists or not. Recent studies have failed to provide convincing evidence that volume, independent of specialization, affects either the incidence of postoperative complications or survival (Kee *et al*, 1999; Parry *et al*, 1999). In contrast, there is increasing evidence that specialisation may be important (Holm *et al*, 1997; Porter *et al*, 1998).

Previous studies have reported differences in outcome among hospitals following surgery for colorectal cancer. Most of these studies have failed to adjust for case-mix and known prognostic factors. Adjustment for the above factors and interpretation of the resultant data is complex; this study illustrates some of the difficulties. Nevertheless, despite these difficulties, there still appear to be differences in outcome among different hospitals. This does not appear to be related to volume but may reflect varying degrees of specialization. Increased specialization and continuing audit are likely to further improve long term outcome following surgery for colorectal cancer.
